# Substrate- and Calcium-Dependent Differential Regulation of Mitochondrial Oxidative Phosphorylation and Energy Production in the Heart and Kidney

**DOI:** 10.3390/cells11010131

**Published:** 2021-12-31

**Authors:** Xiao Zhang, Namrata Tomar, Sunil M. Kandel, Said H. Audi, Allen W. Cowley, Ranjan K. Dash

**Affiliations:** 1 Department of Biomedical Engineering, Medical College of Wisconsin, Milwaukee, WI 53226, USA; tju.zhxiao@gmail.com (X.Z.); ntomar@mcw.edu (N.T.); kandelsunil@gmail.com (S.M.K.); 2 Department of Biomedical Engineering, Marquette University, Milwaukee, WI 53223, USA; said.audi@marquette.edu; 3 Center of Systems Molecular Medicine, Medical College of Wisconsin, Milwaukee, WI 53226, USA; 4 Department of Physiology, Medical College of Wisconsin, Milwaukee, WI 53226, USA

**Keywords:** mitochondrial respiration, ATP synthesis, energy metabolism, substrate utilization, oxidative phosphorylation, respiratory control, calcium regulation

## Abstract

Mitochondrial dehydrogenases are differentially stimulated by Ca^2+^. Ca^2+^ has also diverse regulatory effects on mitochondrial transporters and other enzymes. However, the consequences of these regulatory effects on mitochondrial oxidative phosphorylation (OxPhos) and ATP production, and the dependencies of these consequences on respiratory substrates, have not been investigated between the kidney and heart despite the fact that kidney energy requirements are second only to those of the heart. Our objective was, therefore, to elucidate these relationships in isolated mitochondria from the kidney outer medulla (OM) and heart. ADP-induced mitochondrial respiration was measured at different CaCl_2_ concentrations in the presence of various respiratory substrates, including pyruvate + malate (PM), glutamate + malate (GM), alpha-ketoglutarate + malate (AM), palmitoyl-carnitine + malate (PCM), and succinate + rotenone (SUC + ROT). The results showed that, in both heart and OM mitochondria, and for most complex I substrates, Ca^2+^ effects are biphasic: small increases in Ca^2+^ concentration stimulated, while large increases inhibited mitochondrial respiration. Furthermore, significant differences in substrate- and Ca^2+^-dependent O_2_ utilization towards ATP production between heart and OM mitochondria were observed. With PM and PCM substrates, Ca^2+^ showed more prominent stimulatory effects in OM than in heart mitochondria, while with GM and AM substrates, Ca^2+^ had similar biphasic regulatory effects in both OM and heart mitochondria. In contrast, with complex II substrate SUC + ROT, only inhibitory effects on mitochondrial respiration was observed in both the heart and the OM. We conclude that the regulatory effects of Ca^2+^ on mitochondrial OxPhos and ATP synthesis are biphasic, substrate-dependent, and tissue-specific.

## 1. Introduction

Understanding the molecular basis of how metabolic abnormalities lead to heart and kidney failure is of fundamental importance to advance our understanding of how metabolic regulators may improve the organ’s functions and lessen mortality in disease states such as hypertension [1,2,3,4]. In eukaryotic cells, most of the energy is produced in the form of ATP by mitochondria via oxidative phosphorylation (OxPhos) [5]. Mitochondria also perform many other vital functions, including cation handling and signaling required for maintaining cellular homeostasis [6,7,8,9]. Mitochondrial ATP synthesis is regulated most notably by the availability of ADP and inorganic phosphate (P_i_) [10,11,12,13] and by the availability of substrates [14,15,16]. The latter are required for the optimal functioning of various mitochondrial dehydrogenase (DH) enzymes [17]. These include pyruvate dehydrogenase (PDH), isocitrate dehydrogenase (ICDH), 2-oxoglutarate dehydrogenase (OGDH), and malate dehydrogenase (MDH), which oxidize substrates by transferring protons (H^+^) from organic compounds to an electron acceptor that, in mammals, is either the co-enzyme NAD^+^ (forming NADH) or a flavin co-enzyme (forming FADH_2_) [18]. The activities of these DH enzymes within the tricarboxylic acid (TCA) cycle are known to be differentially regulated via changes in intra-mitochondrial-free calcium (Ca^2+^) concentration [19,20,21,22,23,24,25]. Furthermore, Ca^2+^ is known to have diverse regulatory effects on other mitochondrial transporters and enzymes, including substrate and cation transporters and respiratory complexes [26,27,28]. It is, however, uncertain as to what extent increases in intra-mitochondrial-free Ca^2+^ concentration ([Ca^2+^]_m_)—which occur in parallel with increases in cytosolic-free Ca^2+^ concentration ([Ca^2+^]_c_) [29,30,31]—stimulate the Krebs cycle DH enzymes in situ of key organs such as the kidney and heart, enhancing ATP production during increased workloads and ATP demands, and in response to the availability of different substrates.

It is well known that Ca^2+^ plays a major role in cellular signaling and regulation of mitochondrial function by boosting the production of NADH and FADH_2_ through enhancements in the activities of several mitochondrial substrate transporters and DH enzymes, thereby enhancing mitochondrial respiratory chain activity with a subsequent increase in H^+^ pumping, proton motive force generation, O_2_ consumption, and ATP synthesis [6,32,33,34,35,36]. However, how different respiratory substrates differentially regulate this Ca^2+^-dependent regulation of mitochondrial respiration and ATP synthesis (OxPhos activity) in different tissues having different substrate preferences, such as the heart and kidneys, is not well-understood. In addition, the accumulation of Ca^2+^ within the mitochondrial matrix is known to have detrimental effects on mitochondrial function, specifically OxPhos [6,7,8,9]. Despite the importance of Ca^2+^ in the regulation of mitochondrial respiration and ATP synthesis, it has not been systematically characterized in different tissues, which is the focus of the present study.

[Ca^2+^]_m_ and [Ca^2+^]_c_ are controlled by mitochondrial uptake and release of Ca^2+^ via Ca^2+^ channels and transporters present in the inner mitochondrial membrane (IMM) and by intra-mitochondrial Ca^2+^ sequestration [6,7,8,9,30,37,38,39,40,41]. Specifically, studies using isolated mitochondria have shown that mitochondrial Ca^2+^ uniporter (MCU) and Na^+^/Ca^2+^ exchanger (NCE) pathways and intra-mitochondrial Ca^2+^ sequestration by different Ca^2+^ buffering metabolites and proteins play important roles in Ca^2+^ signaling and [Ca^2+^]_c_ and [Ca^2+^]_m_ regulations [30,37,38,39,40,41,42], thereby regulating mitochondrial energy metabolism. Although the role of Ca^2+^ in excitation–contraction coupling and energy homeostasis in the heart and skeletal muscle has been well studied [32,33,34,35,36,43,44,45], little is known about the extent to which Ca^2+^ participates in kidney bioenergetic function despite having energy requirements second only to the heart [2,46,47,48].

The heart is a constantly active muscle and is the most energy-consuming organ in the human body, requiring high levels of mitochondrial OxPhos activity [49,50,51]. Its huge oxidative metabolism capacity enables up to a six-fold increase in cardiac output during increased workloads and ATP demands without requiring anaerobic metabolism. The kidneys are next to the heart in terms of mitochondrial content and O_2_ consumption at rest [2,46,47]. The kidneys, which have been found to have one of the highest specific metabolic rates among all organs [46,48], must generate enormous energy (ATP) for the reabsorption of great amounts of life-sustaining glomerular filtrates, including electrolytes and various nutrients required to maintain the balance of electrolytes and fluid within the body [1,46,52,53,54].

The impetus for the present study of kidney mitochondrial bioenergetics was the emerging evidence in human and animal models that renal metabolic abnormalities contribute to salt-sensitive hypertension [1,2]. The present study focuses on the kidney outer medulla (OM), which contains thick medullary ascending limbs (mTAL) of the loop of Henle which actively reabsorb ~25% of sodium filtered by the cortical glomeruli [46]. It is a region of the kidney known to be prone to injury in many disease states, such as hypertension [55]. Unlike the kidney cortex, which is overly perfused with blood to sustain high levels of glomerular filtration, blood flow to the kidney OM is quite low (~5–7% of cortical flow) and barely sufficient to supply the O_2_ needed for the transport loads of mTAL; hence, the kidney OM functions in a near-hypoxic state [56]. Blood flow to the vasa recta circulation of the renal medulla must be maintained at low levels to avoid the washout of interstitial osmolytes required to reabsorb water and concentrate the urine while balancing the metabolic needs of mTAL. The reduction of blood flow to the renal medulla is known to reduce its ability to excrete both sodium and water, which results in hypertension [1,57].

Given the contrasting functions of the heart and kidney OM, it is expected that these tissues may utilize different fuels to produce energy (ATP). In contrast to the heart, which largely metabolizes free fatty acids when normally perfused [58,59], the kidney OM is known to largely metabolize glucose via glycolysis-generating pyruvate to be utilized in the TCA cycle and the mitochondrial electron transport chain (ETC) for the production of ATP [1]. Beyond this general knowledge, however, the mitochondrial metabolic pathways and related bioenergetic functions have not been systematically studied in the kidney OM. The extent to which changes in [Ca^2+^]_m_ play a role in the stimulation or inhibition of mitochondrial respiration and ATP synthesis in the kidney OM, compared to the heart, is largely unknown. Even in the heart and skeletal muscle, where Ca^2+^ stimulation of mitochondrial respiration and ATP synthesis are well-recognized at low levels of [Ca^2+^]_m_ [23,60,61], consensus is lacking regarding the quantitative relevance and the sensitivity of the mitochondrial DH enzymes to changes in [Ca^2+^]_m_ and the effect of such changes on mitochondrial respiration and ATP synthesis characterizing OxPhos activity [33]. This may be a consequence of differences in the selection of respiratory substrates used in these isolated mitochondria studies, and differences in the methods used to energize mitochondria or perturb [Ca^2+^]_m_ [32,62,63,64].

The present study was carried out to determine the extent to which mitochondrial respiration and ATP synthesis (OxPhos activity) is substrate- and tissue-dependent, and to determine the extent to which Ca^2+^ can stimulate OxPhos in mitochondria isolated from the heart compared to that from the kidney OM of Sprague–Dawley (SD) rats. ADP-induced mitochondrial respiration was determined at different concentrations of CaCl_2_ in the presence of a variety of respiratory substrates. The results showed that mitochondrial respiration and ATP synthesis are dependent on both the respiratory substrates used and the concentration of CaCl_2_, and that this dependency is tissue-specific and biphasic with respect to the added [Ca^2+^]. These results strengthen our understanding of tissue-specific differences in mitochondrial bioenergetics and regulations, which, in turn, provide a basis for a deeper understanding of the roles of mitochondrial dysfunction in the pathogenesis of chronic diseases, such as hypertension. These results are also useful for building predictive mathematical models of mitochondrial bioenergetics and regulations in the heart and kidney, and to study the emergent properties of cardiac and renal metabolic systems.

## 2. Materials and Methods

The chemicals used in this study were purchased from Sigma–Aldrich (St. Louis, MO, USA) unless otherwise stated. Adult male Sprague–Dawley (SD) rats (8–9 weeks old; 300–350 gm weight) were purchased from Envigo (Madison, WI, USA) and were housed in our animal facilities for at least a week for acclimation to the new environment before being used in this study. Rats were housed in individual cages maintained at 21 ± 2 °C with a 12:12-h of light–dark cycle, and were given water and food ad libitum. The Medical College of Wisconsin (MCW) Institutional Animal Care and Use Committee (IACUC: AUA00000851) approved the animal use and the experimental protocols described below.

### 2.1. Mitochondrial Isolation

Mitochondria were freshly isolated from the ventricles of the heart and the OM of the kidneys of adult SD rats (8–9 weeks, 300–350 gm), as detailed in our recent study [65]. Following an intraperitoneal injection of pentobarbital to achieve a deep plane of anesthesia, the hearts and kidneys were excised, and the mitochondria were isolated, as briefly described below.

#### 2.1.1. Heart Mitochondria

Cardiac mitochondria were isolated via the differential centrifugation method, as described previously [10,65,66,67]. Briefly, the ventricles of the heart were isolated and immediately placed in ice-cold isolation buffer (IB) containing 200 mM mannitol, 50 mM sucrose, 5 mM KH_2_PO_4_, 5 mM MOPS, 1 mM EGTA, and 0.1% BSA, with the pH adjusted to 7.15 with KOH. They were then minced, 2.5 mL of a 5 U/mL protease solution was added, and they were then homogenized for 40 s. The homogenate was first centrifuged twice at 8000× *g* for 10 min at 4 °C to remove the protease. The supernatant was then discarded, and the pellet was re-suspended in the IB to 25 mL and centrifuged at 700× *g* for 10 min. Then, the mitochondria-enriched supernatant was centrifuged at 8000× *g* for 10 min. The resulting mitochondrial-enriched pellet was re-suspended in IB and kept on ice. The mitochondrial protein content was measured using BSA as the standard with Biorad Quick Start Bradford Assay Kit (Biorad, Hercules, CA, USA). Depending on the protein content, the suspended pellet volume was adjusted to have a protein concentration of 12.5 mg protein/mL to allow comparison across groups or conditions without the confounding influence of differences in the concentration of mitochondria loaded into the chamber.

#### 2.1.2. Kidney Outer Medulla (OM) Mitochondria

Renal OM mitochondria were isolated through the differential centrifugation method with a slight modification from that for the cardiac mitochondria [65]. Briefly, OM were dissected from both kidneys, weighed, and minced in the 4 °C IB. The volume was adjusted to a total of 15 mL with IB and centrifuged at 600× *g* at 4 °C for 10 min, and the remaining supernatant was centrifuged at 12,000× *g* for 15 min. The resulting pellet was mixed well in IB and the volume adjusted up to 15 mL. This suspension was centrifuged again for a third time at 12,000× *g* for 15 min, and the final mitochondrial pellet was then transferred to a dark Eppendorf tube, and placed on ice to determine protein content, as described above, to enable adjustments of the final suspension volume to attain a protein concentration of 12.5 mg protein/mL IB.

### 2.2. Mitochondrial O_2_ Consumption Measurement 

**Figure 1:**
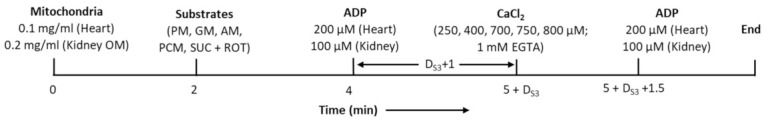
Timeline of the experimental protocol (in minutes) for isolated heart and kidney OM mitochondrial respiration experiments under different metabolic substrates at various Ca^2+^ concentrations. The mitochondria were added at *t* = 0 min and the substrates and ADP were added at *t* = 2 min and *t* = 4 min, respectively. Different concentrations of Ca^2+^, ranging from 250 µM to 800 µM, were added 1 min after reaching steady state 4 respiration, and this was followed by the addition of the same ADP concentration as the first ADP perturbation. D_S3_: state 3 duration for the first ADP addition. PM: pyruvate + malate, GM: glutamate + malate, AM: alpha-ketoglutarate + malate, PCM: palmitoyl-l-carnitine + malate, SUC + ROT: succinate + rotenone.

Mitochondrial O_2_ consumption (respiration) was measured using an Oxygraph-2k (O2k) high-resolution respirometer (Oroboros Instruments, Innsbruck, Austria) with its DatLab 7 software used for data acquisition and analysis. Figure 1 shows the timeline of the experimental protocol for the respiration measurements for all reported studies. Before each experiment, O_2_ concentration in the KCl-enriched respiration buffer (RB) was equilibrated for more than 10 min with the air within the O2k chambers at 37 °C, until a steady stable signal was obtained at an O_2_ concentration of ~205 μM. The experiments were initiated at time *t* = 0 min with isolated mitochondria (0.1 mg protein/mL for heart and 0.2 mg protein/mL for kidney OM) suspended at 37 °C in 2 mL of RB containing (in mM) 130 KCl, 5 K_2_HPO_4_, 20 MOPS, 1 EGTA, and 0.1% BSA, at pH 7.2 adjusted with KOH. The mitochondrial suspension in the RB was continuously stirred (750 rpm) inside the O2k chambers, and chemicals were added to the chamber through the titanium injection port of the stoppers using Hamilton syringes.

In the first set of experiments, state 2 respiration was initiated with the complex I substrates pyruvate + malate at saturating concentrations (PM; 5 + 2.5 mM) at time *t* = 2 min. State 3 respiration was measured by adding 500 and 250 μM ADP for heart and kidney OM mitochondria, respectively, at time *t* = 4 min, and state 4 respiration was measured after phosphorylation of the added ADP to ATP. The functional integrity and intactness of isolated mitochondria on a given experimental day was determined using respiratory control ratio (RCR), defined as the ratio of maximum O_2_ consumption measured after the addition of ADP (state 3) to the state 2 O_2_ consumption (i.e., steady O_2_ consumption after addition of the substrate PM to the O2k chamber containing the mitochondria).

In the second set of experiments, the time-courses of states 2, 3, and 4 respiration were monitored by adding a variety of respiratory substrates (Table 1) to isolated mitochondria (0.1 mg protein/mL for heart and 0.2 mg protein/mL for kidney OM) [65], including pyruvate + malate (PM; 5:2.5 mM), glutamate + malate (GM; 5:2.5 mM), alpha-ketoglutarate + malate (AM; 5:2.5 mM), palmitoyl-l-carnitine + malate (PCM; 0.025:2.5 mM), and succinate + rotenone (SUC + ROT; 10 mM + 1 μM) at time *t* = 2 min, and ADP (200 and 100 μM for heart and kidney OM mitochondria; respectively) at time *t* = 4 min, which initiates state 3 respiration proceeding to state 4 respiration after the conversion of the added ADP to ATP. To assess the effect of Ca^2+^ on mitochondrial O_2_ consumption, various concentrations of Ca^2+^ (250, 400, 700, 750, or 800 μM of CaCl_2_ in the presence of the Ca^2+^ chelator EGTA at 1 mM; negligible changes in total Cl^−^ concentration of 130 mM) were added a minute after state 4 was reached. This was followed by the addition of ADP at the above concentration. O_2_ consumption rates (OCR; JO_2_) during different states of respiration were computed as the negative time derivative of O_2_ concentration measured in the closed O2k chambers and expressed as nmol of O_2_/min/mg of mitochondrial protein. Data were acquired at every 1 s, and 5 data point averages were made to compute the slope of the O_2_ concentration data using the DatLab 7 software version 7.4.0.4. The unique timeline protocol of Figure 1 enabled us to study both the effects of various respiratory substrates and a range of CaCl_2_ concentrations on mitochondrial respiration and ATP synthesis characterizing OxPhos activity.

### 2.3. Control of Extra-Mitochondrial free Ca^2+^ Concentrations

Free Ca^2+^ concentration ([Ca^2+^]_free_) in RB was controlled with 1 mM EGTA. The addition of the buffer components did not alter the total Ca^2+^ concentration ([Ca^2+^]_total_) added to the buffer to achieve the desired [Ca^2+^]_free_. For a given [Ca^2+^]_total_, [Ca^2+^]_free_ was calculated using the Ca-EGTA online calculator v1.3, along with constants from Schoenmakers’s Chelator from Fheuvenet et al. [68] for 1 mM EGTA and other experimental buffer constituents (ionic strength 0.17, pH 7.2, temperature 37 °C). The correspondence between [Ca^2+^]_total_ and [Ca^2+^]_free_ is given in Table 2, which is 0 µM ↔ 0 nM, 250 µM ↔ 100 nM, 400 µM ↔ 200 nM, 700 µM ↔ 750 nM, 750 µM ↔ 900 nM, and 800 µM ↔ 1.2 µM. The calculated [Ca^2+^]_free_ values are subjected to 5% uncertainty, due to a 1% uncertainty in the stability constants, or 0.01 pH unit uncertainty in the measured pH.

### 2.4. Data Analysis and Statistics

All data were analyzed using computer programs written in MATLAB that access the raw data of specific variables from the Excel^®^ spreadsheets of day-to-day experiments generated from the Datlab software. The programs performed the required calculations to calibrate absolute values and changes in variables from one state to another, including statistical analyses (i.e., computations of mean, standard deviation, and standard error of a particular variable over multiple-day experiments). The final data of a particular variable were expressed as mean ± standard error (SE) over at least 3 biological replicates of the same variable (*n* > 3), each measured with two technical replicates. Comparisons within and between groups were performed by ANOVA (analysis of variance) with Tukey’s post hoc test for significance of means.

To characterize the biphasic behavior of the relationship between mitochondrial O_2_ consumption rates (OCR; JO_2_) and free Ca^2+^ concentration ([Ca^2+^]_free_), the following empirical Equation (1) was applied to describe the state 3 JO_2_ − [Ca^2+^]_free_ relationship:

(1) $${\mathrm{JO}}_{2}=\left[V_{0}+\left(V_{\max}{-V}_{0}\right)\left(\frac{{\left[{\mathrm{Ca}}^{2+}\right]}_{\mathrm{free}}}{K_{\mathrm{Ca}}+{\left[{\mathrm{Ca}}^{2+}\right]}_{\mathrm{free}}}\right)\right]\left[\frac{K_{\mathrm{Ca}}{}^{\mathrm{nH}}}{K_{\mathrm{Ca}}{}^{\mathrm{nH}}+{\left[{\mathrm{Ca}}^{2+}\right]}_{\mathrm{free}}{}^{\mathrm{nH}}}\right]$$

where K_Ca_ is the apparent binding constant for Ca^2+^; V_0_ and V_max_ are the basal and maximal state 3 respiratory rates, respectively; nH is the Hill coefficient for inhibitory term, which was set at an optimal value of 3 with several trials of the nonlinear regression fitting of the model to the data. The first term of this equation accounts for the stimulatory effect and the second term accounts for the inhibitory effect of Ca^2+^. V_0_ corresponds to state 3 JO_2_ when [Ca^2+^] is 0.

Experimental data for each group (5 substrates and 2 tissues) were separately fitted to the above kinetic model using the MATLAB curve-fit function “lsqcurvefit” to estimate the values of V_0_, V_max_, K_Ca_, peak Ca^2+^-stimulated state 3 JO_2_, and [Ca^2+^]_free_, necessary to achieve the peak Ca^2+^ stimulated state 3 JO_2_. The resulting residuals and Jacobian matrix were fed to the MATLAB function “nlparci” to calculate the 95% confidence interval for each of the parameters in Equation (1).

## 3. Results

First, we determined the substrate-dependent respiratory rates under different states using isolated heart and kidney OM mitochondria. Five different substrate combinations were used in the study, namely, PM, GM, AM, PCM, and SUC + ROT. After that, we determined the extent and the nature of Ca^2+^ stimulation of mitochondrial respiration and ATP synthesis characterizing OxPhos activity in the heart and kidney OM and the extent of their dependency on the choice of substrates. The first step was to measure the respiratory control ratio (RCR; state 3/state 2 OCR) with the PM substrates with saturated ADP concentration (500 and 250 μM for the heart and kidney OM mitochondria, respectively) to ensure the functional integrity of the isolated mitochondria, which averaged 10.40 ± 0.24 for the heart and 8.54 ± 0.25 for the kidney OM.

**Figure 2:**
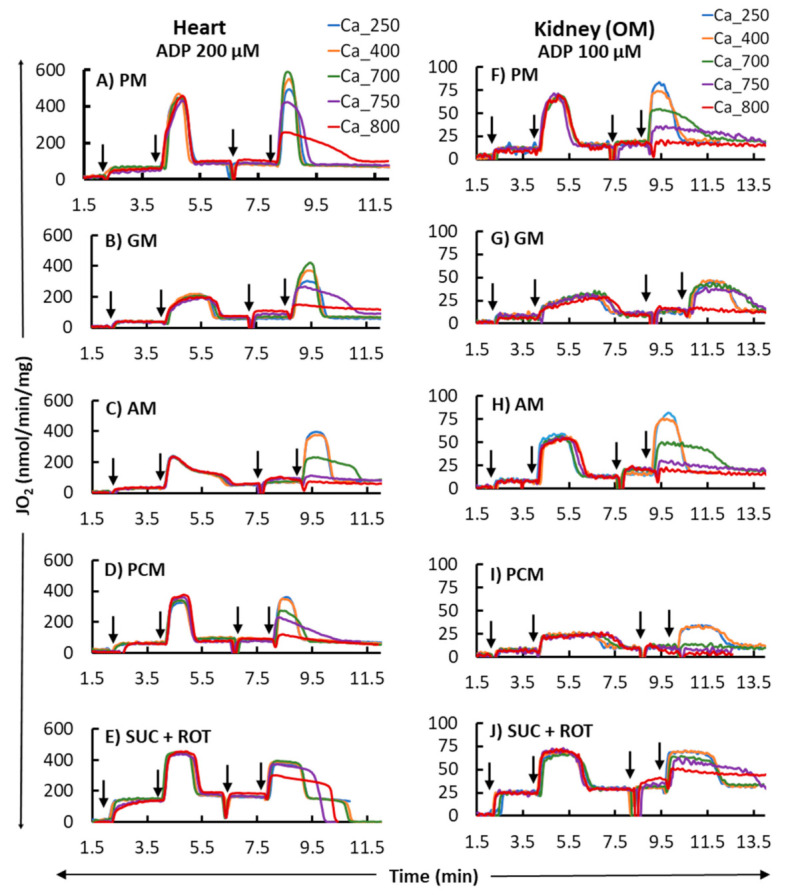
Representative time courses of mitochondrial O_2_ consumption rates (OCR; JO_2_) for different respiratory substrates (PM, GM, AM, PCM, and SUC + ROT) in the heart (left panel; (**A**–**E**) and kidney OM (right panel; (**F**–**J**). States 2, 3, and 4 OCR were monitored by adding the substrates at *t* = 2 min and ADP (200 and 100 µM final concentration for heart and kidney OM mitochondria, respectively) at *t* = 4 min, which initiated state 3 respiration proceeding to state 4 respiration after the conversion of the added ADP to ATP. Different concentrations of CaCl_2_ (250, 400, 700, 750, and 800 µM) in the presence of 1 mM Ca^2+^ chelator EGTA were added in parallel experiments approximately 1 min after reaching the state 4 respiration, and this was followed by the addition of another ADP with the same concentration as used above. The times at which the reagents were added to the mitochondrial suspension are indicated by the vertical arrows.

Figure 2 shows the representative time courses of mitochondrial O_2_ consumption rates (respiration) for the heart and kidney OM for different substrates (PM, GM, AM, PCM, and SUC + ROT), based on the timeline experimental protocol of Figure 1. These data exemplify the dynamic profiles of: (1) tissue-specific differential effects of different substrates on mitochondrial respiration transitioning from state 2 to state 3 to state 4 respirations, and (2) tissue-specific and substrate-dependent differential effects of different [Ca^2+^] on mitochondrial OxPhos (ADP-induced state 3 respiration and ATP synthesis). Importantly, the analysis of mitochondrial respiration with the second dose of ADP demonstrates that the effect of Ca^2+^ on mitochondrial state 3 respiration and ATP synthesis (OxPhos activity) is biphasic in the presence of all substrates studied, except for SUC + ROT, with a stimulatory effect at lower [Ca^2+^] and inhibitory effect at higher [Ca^2+^]. This is characterized by increased peak state 3 respiration and decreased state 3 duration of ADP phosphorylation at low [Ca^2+^] and decreased peak state 3 respiration and increased state 3 duration of ADP phosphorylation at high [Ca^2+^]. Interestingly, for the SUC + ROT substrate in both the heart and kidney OM mitochondria, and for the PCM substrate in the kidney OM mitochondria, only an inhibitory effect of Ca^2+^ was observed for all concentrations of CaCl_2_ added to the mitochondrial suspension.

**Figure 3:**
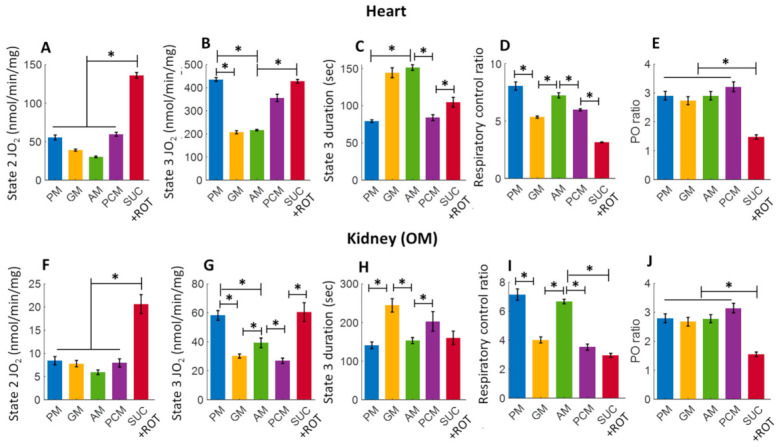
Summary of the different substrate-dependent respiratory parameters derived from the mitochondrial O_2_ consumption rates (OCR; JO_2_) of Figure 2 before adding CaCl_2_ (CaCl_2_ = 0) for the heart (upper panel; (**A**–**E**) and kidney OM (lower panel; (**F**–**J**). (**A**,**F**) State 2 JO_2_; (**B**,**G**) State 3 JO_2_; (**C**,**H**) State 3 duration of JO_2_; (**D**,**I**) Respiratory control ratio (RCR; state 3/state 2 JO_2_); and (**E**,**J**) PO ratio (ratio of ADP consumed to O_2_ consumed). Each bar plot represents the mean value ± SEM (*n* = 4–6). The symbol ‘*’ shows the statistical significance (*p* < 0.05) based on one-way ANOVA with repeated measures.

The bar plots in Figure 3 summarize respiration rates under different states and other relevant respiratory parameters derived from the respiratory responses of different substrates and the first dose of ADP (i.e., 200 and 100 μM for heart and kidney OM mitochondria, respectively) in Figure 2 (no Ca^2+^ addition). Figure 3A,B,F,G show that the extents of states 2 and 3 mitochondrial respiration are substrate-dependent in both heart and kidney OM mitochondria. The orders of substrate-dependent respiration rates are similar for both heart and kidney OM mitochondria, except for the fatty acid-based substrate PCM: the state 3 respiration rate is highest when using PM and SUC + ROT and lowest when using GM and AM as substrates. Interestingly, the state 3 respiration rate in heart mitochondria with PCM substrate was higher than that with GM and AM substrates, but the state 3 respiration in kidney OM mitochondria with PCM substrate was lower than that with GM and AM substrates. Overall, heart mitochondrial state 3 respiration rate is more than eight-fold higher than that of kidney OM mitochondria before the addition of Ca^2+^. Figure 3C,H show the time duration (sec) of state 3 respiration for heart and kidney OM mitochondria for a given ADP concentration. The state 3 respiration time duration was computed as the difference between the time when ADP was added to the isolated mitochondrial suspension and the time when all the added ADP was utilized by the mitochondria (start of state 4). The time duration of the state 3 respiration is usually inversely related to the state 3 respiration rate, i.e., when the state 3 respiration rate is higher, mitochondria would take less time to consume all the added ADP, and vice versa. The other derived respiratory parameters RCR (respiratory control ratio, defined as the ratio of state 3 to state 2 respiration rate) and PO ratio (defined as the ratio of amount of ADP consumed to amount of O_2_ consumed) are shown in Figure 3D,E,I,J. The RCR was slightly higher in heart mitochondria compared to kidney OM mitochondria for a given substrate. In addition, RCR with PM and AM substrates were significantly higher than those with other substrates in both heart and kidney OM mitochondria. The PO ratio was consistently lower than 3 with complex I substrates and approximately 1.5 with a complex II substrate (SUC + ROT).

**Figure 4:**
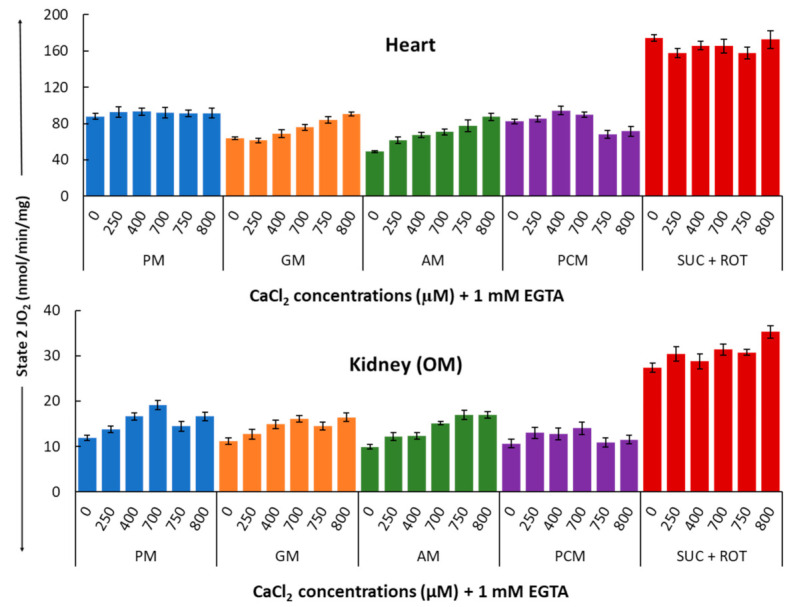
Summary of the different respiratory substrate-dependent effects of Ca^2+^ on mitochondrial state 2 O_2_ consumption rates (OCR; JO_2_) for the heart (upper panel) and kidney OM (lower panel). The X-axis shows CaCl_2_ added to the buffer in the presence of 1 mM Ca^2+^ chelator EGTA. Each bar plot represents the mean value ± SEM (*n* = 4–6) of state 2 OCR.

Figure 4 shows a summary of the substrate-dependent Ca^2+^ effects on the heart (upper panel) and kidney OM (lower panel) mitochondrial state 2 respiration (leak respiration). Heart mitochondria exhibited no change in state 2 respiration at different concentrations of Ca^2+^ in the presence of PM substrates. In contrast, kidney OM mitochondria exhibited a progressive but not significant increase for added Ca^2+^ concentrations between 0 and 700 µM. Among the different substrate combinations studied, state 2 respiration rates with GM and AM substrates show small Ca^2+^-stimulated increases in both heart and kidney OM mitochondria over the range of the Ca^2+^ concentrations studied. This is consistent with the fact that these substrates are involved with Ca^2+^-sensitive metabolic pathways due to the higher activation of alpha-ketoglutarate dehydrogenation (AKGDH) compared to pyruvate dehydrogenase (PDH). The effects of Ca^2+^ concentration on state 2 respiration rates are relatively small in the presence of the substrate combinations PCM and SUC + ROT in both heart and kidney OM mitochondria, indicating that state 2 respiration is independent of Ca^2+^ in the presence of fatty acid-based or complex II-based substrates.

**Figure 5:**
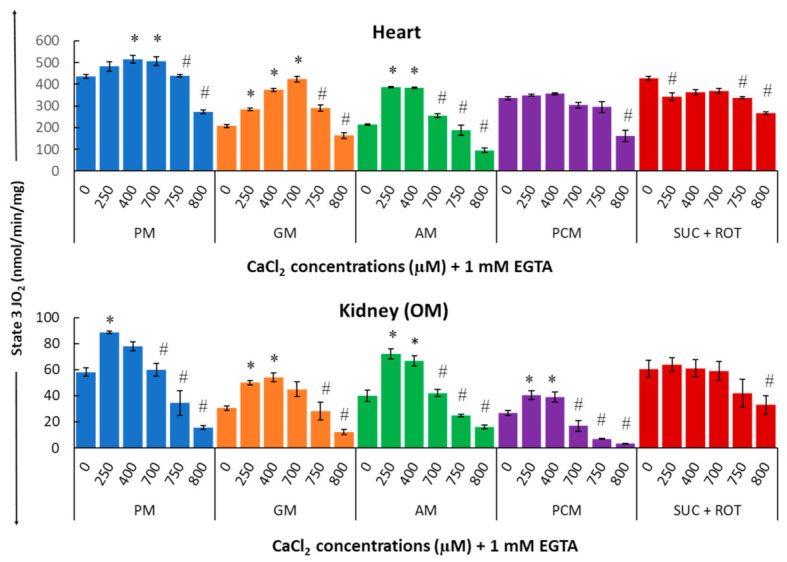
Summary of the different respiratory substrate-dependent effects of Ca^2+^ on mitochondrial state 3 O_2_ consumption rate (OCR; JO_2_) for the heart (upper panel) and kidney OM (lower panel). The X-axis shows CaCl_2_ added to the buffer in the presence of 1 mM Ca^2+^ chelator EGTA. Each bar plot represents the mean value ± SEM (*n* = 4–6) of state 3 OCR. The symbol ‘*’ represents significantly higher results than the baseline state 3 OCR (*p* < 0.05), while the symbol ‘#’ represents significantly lower results than the peak state 3 OCR (*p* < 0.05); both are based on one-way ANOVA with repeated measures.

Figure 5 summarizes the substrate-dependent effects of different concentrations of Ca^2+^ on mitochondrial state 3 respiration in the heart (upper panel) and kidney OM (lower panel). Figure 5 shows that Ca^2+^ differentially regulates mitochondrial state 3 respiration and that this regulatory effect is both substrate-dependent and tissue-specific. In cardiac mitochondria with PM substrates, an ~18% increase in maximal state 3 respiration (JO_2_) was observed with 400 μM [Ca^2+^]_total_ (i.e., 200 nM [Ca^2+^]_free_). For the same substrates, the effect of Ca^2+^ was significantly larger in kidney OM mitochondria, with an ~52% increase in maximal state 3 JO_2_ achieved with only 250 μM [Ca^2+^]_total_ (i.e., 100 nM [Ca^2+^]_free_). Interestingly, with GM substrates, ~105% and ~77% increases in maximal state 3 JO_2_ were observed with 700 μM and 400 μM [Ca^2+^]_total_ (i.e., 720 nM and 200 nM [Ca^2+^]_free_) for heart and kidney OM mitochondria, respectively. In contrast, with AM substrates, only 250 μM [Ca^2+^]_total_ (i.e., 100 nM [Ca^2+^]_free_) was sufficient to stimulate ~80% increase in maximal state 3 JO_2_ in both heart and kidney OM mitochondria. The effect of Ca^2+^ on state 3 JO_2_ with PCM substrates was insignificant in cardiac mitochondria, but quite prominent (~51% increase) in kidney OM mitochondria. Finally, Figure 5 also shows that in both heart and kidney OM mitochondria, with SUC as the substrate in the presence of ROT, the addition of Ca^2+^ consistently resulted in an inhibitory effect on the state 3 respiratory rate.

Indeed, for all complex I substrates studied, (PM, GM, and AM), a biphasic relationship is observed in which a small increase in [Ca^2+^] has a stimulatory effect on state 3 respiration, whereas a large increase in [Ca^2+^] has an inhibitory effect. Moreover, the concentrations of Ca^2+^ necessary for this switching from the stimulatory to inhibitory effects are both substrate-dependent and organ-specific. For example, in the presence of PM substrates, state 3 respiration in heart mitochondria peaked at 400 µM [Ca^2+^]_total_, as compared to 250 µM [Ca^2+^]_total_ in kidney OM mitochondria. When using PCM as a substrate combination, the addition of Ca^2+^ induced an increase in state 3 respiration in kidney OM mitochondria for concentrations between 0 and 400 μM. However, this effect was negligible in heart mitochondria. When using SUC as a substrate in the presence of ROT, the addition of Ca^2+^ induced a partial inhibition of state 3 respiration for most of the [Ca^2+^] studied in heart mitochondria, but only for the highest [Ca^2+^] studied in kidney OM mitochondria. Our results also show that, except for the substrate combination SUC + ROT, the effects of varying [Ca^2+^] on mitochondrial state 3 respiration and ATP synthesis are more prominent in kidney OM mitochondria than those in heart mitochondria.

**Figure 6:**
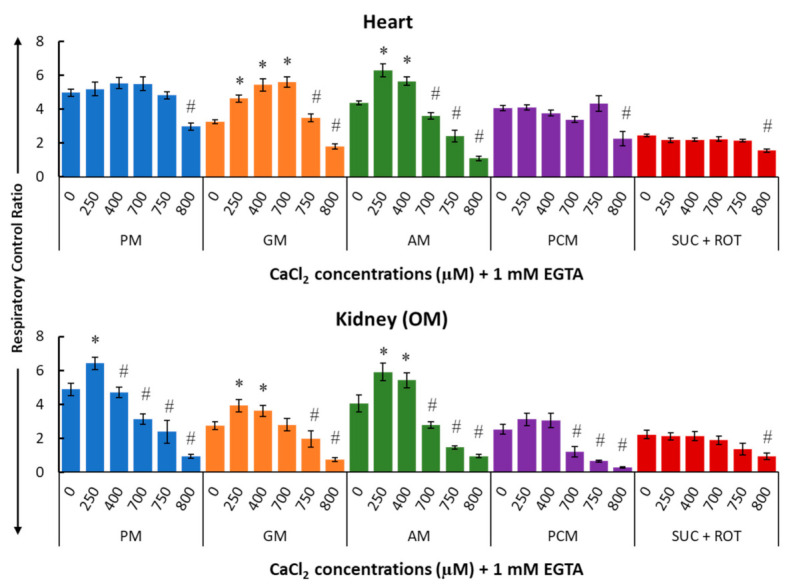
Summary of the different respiratory substrate-dependent effects of Ca^2+^ on the respiratory control ratio (RCR; state 3/state 2 OCR) for the heart (upper panel) and kidney OM (lower panel) mitochondria. The X-axis shows CaCl_2_ added to the buffer in the presence of 1 mM Ca^2+^ chelator EGTA. Each bar plot represents the mean value ± SEM (*n* = 4–6) of RCR. The symbol ‘*’ denotes significantly higher results than the baseline RCR (*p* < 0.05), while the symbol ‘#’ denotes significantly lower results than the peak RCR (*p* < 0.05); both are based on one-way ANOVA with repeated measures.

Figure 6 summarizes the effects of Ca^2+^ on the respiratory control ratio (RCR; ratio of the state 3 to state 2 respiration rate) for different substrate combinations in isolated mitochondria from the heart (upper panel) and kidney OM (lower panel). The RCR data show a biphasic pattern similar to that for state 3 respiration (Figure 5) since the state 2 respiration was fairly constant (Figure 4).

**Figure 7:**
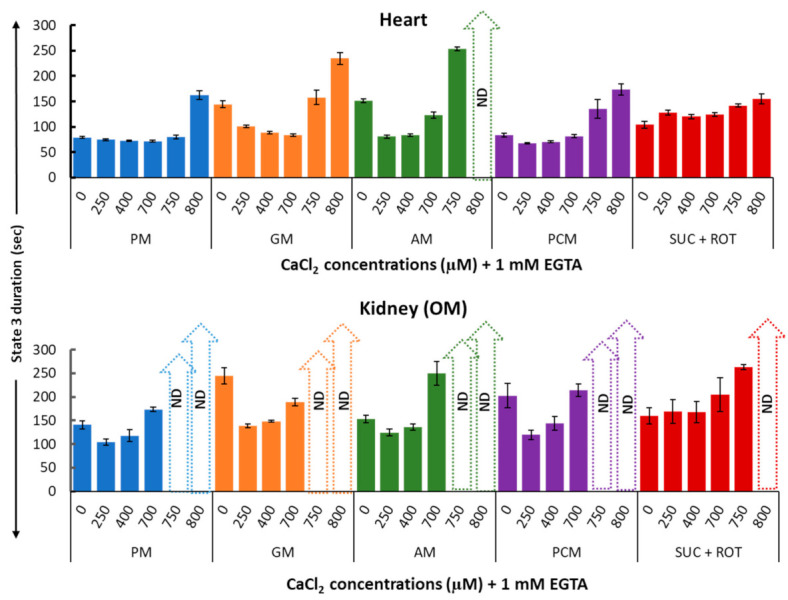
Summary of the different respiratory substrate-dependent effects of Ca^2+^ on the heart (upper panel) and kidney OM (lower panel) mitochondrial state 3 duration of OCR. The X-axis shows CaCl_2_ added to the buffer in the presence of 1 mM Ca^2+^ chelator EGTA. Each bar plot represents the mean value ± SEM (*n* = 4–6) of state 3 durations of OCR. ND: not determined.

Figure 7 provides a summary of the effects of varying [Ca^2+^] on the duration of mitochondrial state 3 JO_2_ (in seconds) in the presence of different substrate combinations for both heart (upper panel) and kidney OM (lower panel). These data suggest that small increases in [Ca^2+^] resulted in higher state 3 JO_2_ peaks and shorter state 3 durations, whereas large increases in [Ca^2+^] resulted in lower state 3 JO_2_ peaks and longer state 3 durations as compared to values with no [Ca^2+^] added. In general, the duration of state 3 JO_2_ is inversely proportional to peak state 3 JO_2_. This is due to the fact that if the respiration is slower, it would take more time for mitochondria to convert the added ADP to ATP. The white strips with upward arrows in Figure 7 highlight the values that are not quantitatively defined (ND) and cannot be calculated because higher concentrations of Ca^2+^ greatly inhibited state 3 JO_2_, preventing an accurate determination of the state 3 duration.

**Figure 8:**
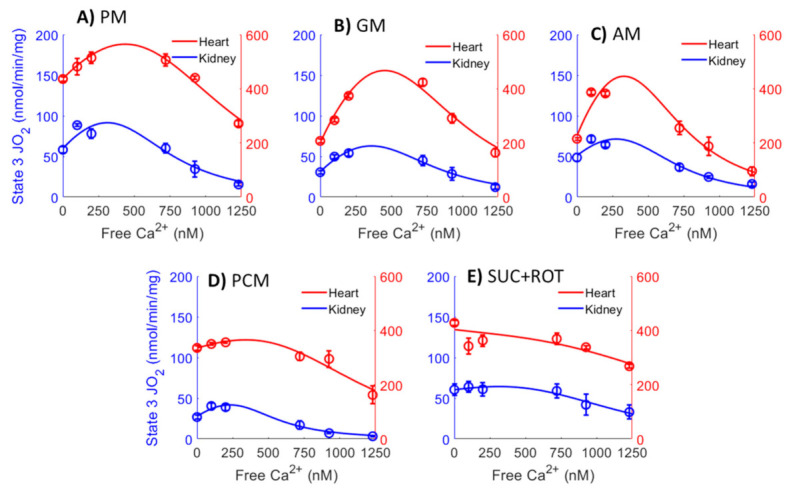
Comparison of the mitochondrial state 3 O_2_ consumption rates (OCR; JO_2_) data between the heart (red points) and kidney OM (blue points) for the different respiratory substrate combinations (**A**) PM, (**B**) GM, (**C**) AM, (**D**) PCM, and (**E**) SUC + ROT) used in the study. Solid lines are the nonlinear regression fits of Equation (1) to the data. The X-axis shows [Ca^2+^]_free_ in the extra-mitochondrial buffer, which is calculated from the added CaCl_2_ ([Ca^2+^]_total_) and 1 mM Ca^2+^ chelator EGTA based on Table 2.

A detailed data analysis using nonlinear regression was performed to determine the relationships between [Ca^2+^]_free_ and state 3 JO_2_ with different substrate combinations. The data in Figure 8 (circle with error bars) are the same as state 3 JO_2_ data in Figure 5, except that the total Ca^2+^concentrations ([Ca^2+^]_total_) was converted to the free Ca^2+^ concentration ([Ca^2+^]_free_) present in the extra-mitochondrial buffer space, based on the Ca^2+^ chelator calculator [68], as shown in Table 2. Solid lines are the nonlinear regression fits of Equation (1)’s solutions to the data. Nonlinear regression was performed as described in the Methods section. This nonlinear regression analysis enabled us to obtain more accurate estimates of the peak Ca^2+^-stimulated state 3 JO_2_ and [Ca^2+^]_free_ required for achieving this peak Ca^2+^ stimulated state 3 JO_2_ for ATP synthesis than those inferred from the data in Figure 5. It is apparent from Figure 8 that the JO_2_ − [Ca^2+^]_free_ curves for the heart and kidney OM mitochondria are almost the scaled versions of each other for all substrate combinations studied, except for the substrate combinations of GM and AM, where the heart mitochondria curve showed a steeper decline at higher [Ca^2+^]_free_. This indicates that heart mitochondria are more sensitive to Ca^2+^ inhibition when GM or AM are provided as substrate combinations.

**Figure 9:**
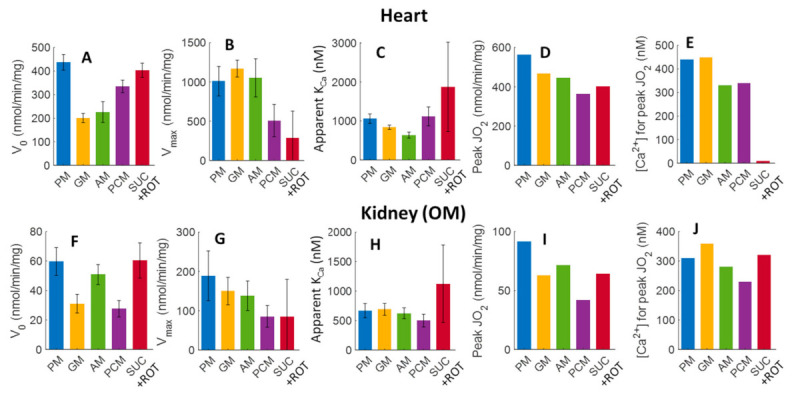
Estimated values of V_0_, V_max_, apparent K_Ca_, peak Ca^2+^ stimulated state 3 JO_2_, and [Ca^2+^]_free_ required for achieving the peak Ca^2+^ stimulated state 3 JO_2_, and their corresponding 95% confidence intervals obtained based on the nonlinear regression (fitting) of Equation (1) and the state 3 JO_2_ data from Figure 5. Bar plots in the upper panels (**A**–**E**) and bottom panels (**F**–**J**) represent the parameters for the heart and kidney OM mitochondria, respectively, for different respiratory substrates (PM, GM, AM, PCM, and SUC + ROT). The peak Ca^2+^ stimulated state 3 JO_2_ (**D**,**I**) and [Ca^2+^]_free_ required for achieving the Ca^2+^ peak stimulated state 3 JO_2_ (**E**,**J**) for each substrate and tissue was predicted by the fitting.

The estimated parameter values, including V_0_, V_max_, apparent K_Ca_, peak Ca^2+^ stimulated state 3 JO_2_, and [Ca^2+^]_free_, required to achieve this peak, along with the corresponding confidence intervals (represented as error bars) are shown in Figure 9. V_0_ values (state 3 JO_2_ at [Ca^2+^]_free_ = 0) are highest for the substrate combinations SUC+ROT and PM for both heart and kidney OM mitochondria. In general, V_0_, V_max_, and peak Ca^2+^-stimulated state 3 JO_2_ values for heart mitochondria are several folds higher than those for kidney OM mitochondria. However, the apparent binding constants (K_Ca_ values; nM) and [Ca^2+^]_free_ required for achieving the peak Ca^2+^ stimulated state 3 JO_2_ for mitochondria from heart and kidney OM are of the same order of magnitude.

## 4. Discussion

Mitochondrial metabolism is known to play an important role in many human disorders including heart failure, hypertension, diabetes, cancer, brain disorders, and many others [3,4,69]. It is remarkable that relatively few studies have focused on the role of renal mitochondrial metabolism despite the close link between renal tubular transport activities and the critical regulation of fluid and electrolyte homeostasis required for survival [1]. The mitochondrial bioenergetic system is a complex network that, within varying organs/tissues, is influenced by energetic needs, the source and availability of respiratory substrates for intermediate metabolism [53,64], unique transporters of these substrates [46,53], different expression and activity of enzymes, and variations in the key determinants of the activity of these enzymes such as Ca^2+^ and Mg^2+^ [21,24]. The bioenergetic aspects of mitochondrial functions have been challenging, and reports of studies even of the same organ, such as the heart, have been conflicting since many key factors that influence ATP production have not been consistently controlled or quantitatively accounted for in most studies [5,10,15,35,61,63]. In the present study, we demonstrated that different respiratory substrates resulted in dramatically different respiratory rates in the heart and kidney OM mitochondria, consistent with our recent study [65]. In addition, the effects of Ca^2+^ upon mitochondrial respiration and ATP production in the heart and kidney OM were distinctly dependent on the respiratory substrates [63]. The respiratory parameters of the heart and kidney OM mitochondria in our study were quantitatively determined under highly controlled conditions, with mitochondria respiring in the presence of different respiratory substrates. The effects of Ca^2+^ on the heart mitochondrial respiration and ATP production have been extensively studied [32,63,64], but little is known about the effects of Ca^2+^ on kidney mitochondrial bioenergetics and regulations. Although dietary Ca^2+^ is clearly important in a wide range of biological functions, the use of dietary Ca^2+^ supplements in heart and kidney diseases remains controversial. It is well known that kidney disease leads to increased production of the parathyroid hormone and a build-up of phosphate in the body which, in turn, binds to Ca^2+^ and leads to brittle bones [70]. This has led to widespread use of dietary Ca^2+^ supplements, which have been of questionable value in reducing the risk of osteoporosis and may lead to Ca^2+^-based plaque buildup [71]. Remarkably, the effects of dietary Ca^2+^ upon the efficiency of energy production in the heart and kidney are unknown.

Thus, in the present study, we specifically focused on investigating the extent to which Ca^2+^ can stimulate or inhibit OxPhos and ATP synthesis in mitochondria isolated from the heart compared to that from the kidney OM of the same animal, and how this regulatory effect is dependent on respiratory substrates. Mitochondria were isolated from the heart and kidney OM obtained from the same rat to control for environmental factors, diet, and age. As such, an important outcome of the present study is a novel set of data showing that Ca^2+^ regulation of mitochondrial respiration and ATP synthesis is biphasic in both the heart and OM mitochondria and that these effects are both substrate-dependent and tissue-specific.

### 4.1. Intermediate Metabolism and Energy Production: Unique Challenges Due to Different Energy Demands of the Heart and Kidneys

Although the functions of the heart and kidneys are uniquely different, both have enormous energy requirements and are subject to a broad range of stressors. In addition to differences in function, the heart and kidneys have different morphology, structure, and mitochondrial content [2,72,73]. Human kidneys have a metabolic rate estimated to be nearly 400 kcal/kg tissue, a rate similar to that for the resting heart [74]. With strenuous exercise, the human heart may increase its output and O_2_ consumption up to six-fold [58,75,76]. The kidneys of humans subjected to a high salt diet increase their energy usage for tubular reabsorptive activity by an estimated 50% (33.5 to 49.2 kcal/day). This is based on a known stoichiometry of 4.6 for renal transepithelial Na^+^ transport to ATP usage with an assumption of 7.3 kcal/mol for the free energy that is equivalent of ATP [77]. Intermediate metabolism and substrate utilization for the heart and kidneys are known to be different. Heart mitochondria utilize fatty acids as the primary fuel, producing 60–70% of the energy [58,59]. In contrast, the intermediate metabolism and preference of the substrates of the kidney greatly differ between the nephron segments of the cortex and medulla [1]. The proximal tubules (PT) of the cortex rely largely upon lipids, fatty acids, ketone bodies, lactate, and some amino acids. The thick medullary ascending limbs (mTAL) of the medulla utilize primarily glucose, succinate, or lactate [1], with only the glycolysis pathway used when needed to generate pyruvate [1].

The heart is challenged by rapid changes in energy demands from a resting to an exercise state, and the mitochondria of myocytes face great fluctuations of O_2_ consumption and ATP production [21,36,76]. The kidneys, although viewed as being more stable in metabolic demands, can also face large fluctuations in these demands when suddenly faced with a high workload imposed by a large salty meal of carbohydrates and proteins. Unlike the heart, the kidney can be thought of as two separately functioning organs with vastly different blood flows and metabolic demands. The kidney cortex receives a blood flow well in excess of its metabolic needs, which is necessary for the bulk filtration at glomeruli to remove the metabolic wastes. It is over-perfused for its normal metabolic needs, and its partial pressure tissue oxygen levels are ~50 mmHg. By contrast, the kidney OM is metabolically quite similar to the heart in that blood flow and oxygen levels are low (~10 to 15 mmHg) [78,79]. Yet, the metabolic needs of the mTAL, driven by the active reabsorption of ~25% of the total sodium filtered by the cortical glomeruli of the kidney, require sufficient O_2_ delivery and substrates to meet these demands. Similar to the heart, as mTAL of the kidney are faced with increased metabolic needs; greater blood flow is required to prevent a hypoxic state [1,79]. To elucidate the contributions of mitochondrial OxPhos towards ATP production and its regulation by Ca^2+^ to the tissue-specific physiology and energy needs of the heart and kidney OM, we performed a systematic investigation of the regulation of mitochondrial bioenergetics involving different respiratory substrates and different extra-mitochondrial [Ca^2+^]. These results further our understanding of tissue-specific differences in mitochondrial bioenergetics and regulations under physiological and pathophysiological conditions. These results are also useful for building predictive mathematical models of mitochondrial bioenergetics and regulations to investigate the emergent properties of cardiac and renal metabolic systems.

### 4.2. Substrate-Dependent Mitochondrial Respiratory Rates for the Heart and Kidney OM with Addition of a Fixed ADP Concentration

Heart and kidneys have different energy demands, and hence, their mitochondrial OxPhos capacities and efficiencies are expected to be different [80,81]. However, integrated quantitative studies have not been undertaken to shed light on the reasons for the differences in the OxPhos capacities and efficiencies of these organs and tissues. It is scientifically informative to determine the mitochondrial bioenergetics of the heart and kidneys using mitochondria isolated from the same animal [65]. In order to dissect the functionality of OxPhos, we employed different TCA cycle substrates, followed by the addition of a fixed ADP concentration, to isolate mitochondria from the heart and kidney OM. This protocol enabled us to determine and compare tissue- and substrate-specific respiratory states and rates with an identical stimulus. Figure 3 depicts the summary of the substrate- and tissue-specific comparisons of mitochondrial O_2_ consumption rates (JO_2_; OCR) for isolated heart and kidney OM mitochondria following the timeline protocol of Figure 1 with no CaCl_2_ addition. The OCR (states 2 and 3), state 3 durations (sec), RCR, and PO ratios were derived from the dynamic data shown in Figure 2 before the CaCl_2_ addition. The state 2 respiration was significantly higher in the presence of the FADH_2_-linked substrate SUC in the presence of ROT (SUC + ROT), in comparison to the NADH-linked substrates provided to heart and kidney OM mitochondria (Figure 3A,F). In contrast, state 3 respiration was highest in the presence of both PM and SUC + ROT in heart and kidney OM mitochondria (Figure 3B,G). GM is one substrate combination which resulted the lowest state 3 respiration and OxPhos efficiency for the heart and kidney OM mitochondria, and hence, the longest state 3 duration (Figure 3C,H). Moreover, heart mitochondria showed the highest RCR with PM substrates. RCR with SUC + ROT was the lowest for both heart and kidney OM mitochondria. The ratio of the added ADP concentration to the utilized O_2_ concentration in the chamber during the state 3 respiration is called the PO ratio for OxPhos. The PO ratios for the NADH-linked substrates averaged ~2.75, and that for the FADH_2_-linked substrates averaged ~1.5 for heart and kidney OM mitochondria, which is consistent with existing knowledge [65,82,83,84], validating the accuracy of the present studies.

### 4.3. Current Understanding of Mitochondrial Ca^2+^ Regulation and Regulation of Mitochondrial Substrate Transport and Energy Metabolism by Ca^2+^

Since Ca^2+^ plays a major role in regulating mitochondrial OxPhos and ATP synthesis [26,60], it is therefore crucial to understand the major mechanisms that regulate mitochondrial Ca^2+^ concentration, [Ca^2+^]_m_. These include mitochondrial Ca^2+^ influx, efflux, and buffering mechanisms. Mitochondrial Ca^2+^ influx occurs through the electrophoretic Ca^2+^ uniporter (MCU), while Ca^2+^ efflux occurs through the Na^+^-dependent electrogenic 3Na^+^/Ca^2+^ exchanger (NCE) and/or Na^+^-independent electroneutral Ca^2+^/2H^+^ exchanger (CHE) at the inner mitochondrial membrane (IMM) [37,38,39,40,85,86]. In addition, the Ca^2+^ influx via MCU and the Ca^2+^ efflux via NCE in energized mitochondria are driven by membrane potential, (ΔΨ_m_) and the Ca^2+^ influx via MCU is inhibited by cytosolic Mg^2+^ [30,37]. Furthermore, [Ca^2+^]_m_ is controlled by complex mitochondrial Ca^2+^ buffering mechanisms [28]. To maintain steady-state [Ca^2+^]_m_, a dynamic balance is required between the MCU-based Ca^2+^ influx, the NCE- and/or CHE-based Ca^2 +^ efflux, and Ca^2+^ buffering within the mitochondrial matrix [87,88]. The relationship between [Ca^2+^]_c_ to [Ca^2+^]_m_ and the influence of [Ca^2+^]_m_ on the TCA cycle was evaluated in the presence of different ionic concentrations, such as Na^+^ and Mg^2+^, in the buffers by [23,30,31] and modeled by [86,89]. It is important to know that the dominant Ca^2+^ transport system is tissue-specific, with NCE more dominant in heart and brain mitochondria [90], while CHE is more dominant in liver and kidney mitochondria [91,92,93].

The three consequential functional phases of mitochondrial Ca^2+^ can be distinguished, viz. the regulation of mitochondrial DH enzymes, the buffering of extramitochondrial Ca^2+^, and ultimately, the activation of the mitochondrial permeability transition pore (mPTP) [20,29,94,95]. There is ample evidence that isolated mitochondria can buffer extramitochondrial Ca^2+^ to the same extent as mitochondria in vivo [96,97]. Since [Ca^2+^]_m_ is crucial for regulating mitochondrial energy metabolism in response to changes in workload, the Ca^2+^ buffering level in the mitochondrial matrix is critical to reaching the desired steady-state [Ca^2+^]_m_ level. Steady-state [Ca^2+^]_m_ controls mitochondrial ATP production via Ca^2+^ regulation of the mitochondrial DH enzymes that activate mitochondrial metabolic machinery and, in the process, stimulate ATP production to meet the cellular energy demands [25,98]. Therefore, it was assumed earlier that [Ca^2+^]_m_ controls the mitochondrial energy metabolism without considering the role of cytoplasmic Ca^2+^ ([Ca^2+^]_c_) on the mitochondrial substrate transport that ultimately impacts the mitochondrial energy metabolism.

Several studies using MCU knockout mice showed that deleting MCU did not alter OxPhos, suggesting that other factors, in addition to [Ca^2+^]_m_, are responsible for the regulation of OxPhos [99,100,101,102,103]. Substrate transport has a great impact on the ability of Ca^2+^ to regulate metabolic activities [104]. For instance, [Ca^2+^]_c_ stimulates OxPhos to respond to increased cellular workload by adjusting the rate of pyruvate transport from the cytosol to the mitochondria [105]. For this reason, the role of a malate–aspartate shuttle (MAS)-dependent substrate transport in regulating OxPhos responses is crucial. Interestingly, the study by Szibor et al. [99] concluded that [Ca^2+^]_c_ controls ~85% of OxPhos rates when pyruvate is provided as a substrate, and the remaining ~15% is regulated by [Ca^2+^]_m_. The mitochondrial glutamate–aspartate carrier (Aralar), an essential component of MAS with a regulatory Ca^2+^-binding site facing the inter membrane space (IMS), promotes Ca^2+^ sensitivity of the MAS [106,107]. It is interesting to note that the MAS senses the [Ca^2+^]_c_ and not the [Ca^2+^]_m_ [108]. In contrast to the Ca^2+^ activation of DH enzymes, the Ca^2+^ activation of the Aralar carrier occurs by increasing its capacity (i.e., V_max_), rather than by decreasing its affinity for Ca^2+^ (i.e., K_Ca_). Apart from glutamate transport [109], Aralar also transports redox equivalents into the mitochondrial matrix [110,111]. These results suggest that the concept of mitochondrial OxPhos stimulation through intra-mitochondrial Ca^2+^ is not consistent with all existing knowledge since it overlooks the fact that the mitochondrial processes can also be activated by Ca^2+^ ions that are not already accumulated within the mitochondrial matrix. The present study dissects the roles of different extra-mitochondrial [Ca^2+^] on mitochondrial state 3 respiration in heart and kidney OM in the present of the different respiratory substrate combinations as mitochondrial fuel. Our results suggest that Ca^2+^ differentially regulates mitochondrial state 3 respiration, and that this differential regulation is both substrate-dependent and organ-specific (Figure 5). Kidney OM mitochondria demonstrated more sensitivity towards the extra-mitochondrial Ca^2+^ perturbations, resulting in higher state 3 respiration in comparison to heart mitochondria for all substrate combinations studied. Interestingly, in the presence of the FADH_2_-based substrate SUC + ROT, the addition of Ca^2+^ consistently resulted in an inhibitory effect on state 3 respiratory rate for both heart and kidney OM mitochondria. Based on the above-mentioned existing knowledge regarding Ca^2+^ regulation of substrate transport, we can interpret our results as showing that different organs (heart and kidney OM in the present study) may vary in the extent of MAS activation and MCU-mediated Ca^2+^ uptake.

### 4.4. Complex Effects of Respiratory Substrates upon Ca^2+^ Activation of Mitochondrial Respiration and ATP Production in the Heart and Kidney OM

Little is known about whether mitochondria of kidney OM respond to Ca^2+^ and metabolic substrates in a manner similar to that of the heart, or whether they respond differently. The free Ca^2+^ level in cardiac mitochondria is 50–100 nM at rest, but can increase to >1µM during exercise [7,58,63]. It is also known that the level of free Ca^2+^ in the kidney is higher [112].

The present study finds that, although kidney OM mitochondria can retain and buffer less Ca^2+^ than heart mitochondria [112], the effects of Ca^2+^ upon mitochondrial state 3 respiration in the kidney OM are significantly greater than those in the heart. This was particularly observed when PM and PCM were provided as substrate combinations (Figure 5). Specifically, Ca^2+^ induced a significant increase in kidney OM mitochondrial state 3 respiration in the presence of PM and PCM substrate combinations. An energy-linked mitochondrial uptake of Ca^2+^ in the presence of phosphate has been studied by Carafoli et al. [113]. They demonstrated that kidney mitochondria can accumulate Ca^2+^ around 2500 ng-atoms/mg of mitochondrial protein in the presence of ADP. The same study sheds light on the effects of mitochondrial Ca^2+^ accumulation by showing the functional responses of the mitochondria to lower [Ca^2+^], and by showing higher state 3 respiration with Ca^2+^ stimulation than with ADP alone. The results of the present study show that, in comparison to kidney OM, the Ca^2+^-induced increase in heart mitochondrial state 3 respiration was negligible, exhibiting only an ~18% increase with PM and an ~6% increase with PCM as substrate combinations (Figure 5).

Interestingly, as seen in Figure 5, Ca^2+^ has a stimulatory effect on mitochondrial state 3 respiration in the presence of complex I substrates, but not in the presence of complex II substrates. Ca^2+^ enhances both the heart and kidney OM mitochondrial state 3 respiration in the presence of NADH-generating substrates, including PM, GM, or AM. However, no activation was observed in mitochondria of either tissue when SUC was provided as the substrate. This can be explained by the fact that FADH_2_ is generated from complex II upon using SUC as a substrate, so the DH enzymes are mostly inactive and the Ca^2+^ effects are, thereby, minimal. In contrast, palmitoyl-carnitine is a long-chain fatty acid, and its oxidation generates equal molar amounts of NADH and FADH_2_. The magnitude of the Ca^2+^ modulation effects of PCM groups may therefore be expected to fall in between complex I groups (PM, GM, AM) and complex II groups (SUC), as we have observed (Figure 5). In brief, Ca^2+^ has strong activation effects on respiration when complex I substrates are used, and has little to effect when complex II substrates are used. Importantly, the results in this study are consistent with the previous knowledge—that Ca^2+^ activation is largely a consequence of dehydrogenase (DH) enzymes in the TCA cycle—and also provide the first quantitative data of these relationships [114,115].

**Figure 10:**
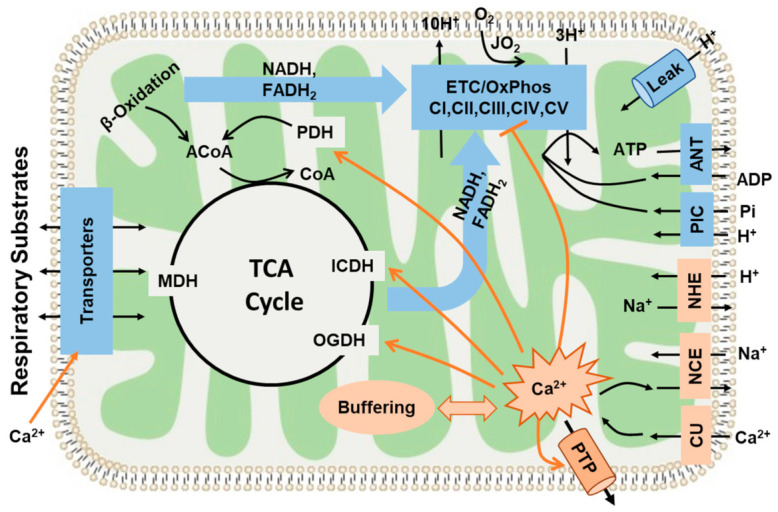
This diagram shows the major pathways and processes regulating mitochondrial bioenergetics and Ca^2+^ homeostasis. It includes mitochondrial substrate transport and oxidation; tricarboxylic acid (TCA) cycle; electron transport chain (ETC), H^+^ pumping, and oxidative phosphorylation (OxPhos); inorganic phosphate carrier (PIC: Pi^−^-H^+^ cotransporter); adenine nucleotide translocase (ANT: ATP-ADP exchanger); and H^+^ leak. It also includes the pathways of mitochondrial Ca^2+^ uptake, release, and buffering regulating mitochondrial Ca^2+^ homeostasis (CU: Ca^2+^ uniporter, NCE: Na^+^/Ca^2+^ exchanger, and NHE: Na^+^/H^+^ exchanger). The diagram also shows the hypothetical regulatory (activation or inhibition) sites of Ca^2+^ on mitochondria OxPhos and ATP synthesis. Ca^2+^ activates mitochondrial dehydrogenase enzymes (PDH: pyruvate dehydrogenase, ICDH: isocitrate dehydrogenase, and OGDH: oxoglutarate dehydrogenase) at low concentrations. However, high concentrations of Ca^2+^ can inhibit mitochondrial complex I and can result in the opening of a mitochondrial permeability transition pore (PTP), leading to mitochondrial dysfunction.

As mentioned previously, mitochondrial energy metabolism is regulated by Ca^2+^ via its effect on major DH enzymes [19,115]. As shown in Figure 10, these enzymes serve as targets for regulation by [Ca^2+^]_m_. The differential regulation of these DH enzymes by Ca^2+^ lies in their different binding activation constants (K_Ca_) for Ca^2+^ and, therefore, these are sensitive to different ranges of [Ca^2+^] [116]. FAD-glycerol phosphate dehydrogenase (GPDH) senses Ca^2+^ in the IMS (K_Ca_ = 0.1 µM) [117]. The other three DH enzymes that sense [Ca^2+^]_m_ are the pyruvate dehydrogenase (PDH) phosphatase (K_Ca_ ~ 1 µM; the complex that modulates the PDH activity), isocitrate dehydrogenase (ICDH), and oxoglutarate dehydrogenase (OGDH). PDH and OGDH are the primary DH enzymes responsible for pyruvate and α-ketoglutarate oxidation, respectively. High mitochondrial [Ca^2+^] stimulates the activity of PDH phosphatase, which further dephosphorylates PDH and increases its activity [98]. The affinity of ICDH for calcium (K_Ca_) ranges from 5 to 50 μM depending on the ATP/ADP ratio [118]. OGDH, a TCA cycle enzyme, catalyzes the conversion of α-ketoglutarate to succinyl-CoA and is also stimulated by Ca^2+^ (K_Ca_ = 1 μM). ICDH and OGDH enzymes become more sensitive to Ca^2+^ with a decrease in the NADH/NAD^+^ ratio [25,119]. The high activation constant (K_Ca_) makes ICDH less relevant since inhibitory effects occur before [Ca^2+^] reaches the K_Ca_ for ICDH. This may explain the relatively moderate activation effects of Ca^2+^ with PM and PCM as substrate combinations in heart mitochondria. The differences in the Ca^2+^ binding activation constants (K_Ca_) also explain the reason behind the substrate- and organ-dependent [Ca^2+^] required to reach maximum respiration. Interestingly, Ca^2+^ also increases the MAS activity [116,120] that activates mitochondrial respiration when using GM as substrates. We can state that this might be the reason for relationships between state 3 respiration and [Ca^2+^], as summarized in Figure 5, which shows the integrated effects of intra-mitochondrial Ca^2+^ on DH enzymes in the TCA cycle and the activation effects of extra-mitochondrial Ca^2+^ on MAS activity [116].

It is evident that the effects of Ca^2+^ upon mitochondrial state 3 respiration are determined by the factors through which the components of the TCA cycle reactions are activated [19,23]. For example, when using AM as a substrate combination, the enzyme PDH is not active in isolated mitochondria and, hence, is less relevant to the activation effect of Ca^2+^. When using PM as a substrate combination, PDH becomes the dominant enzyme that sensitizes mitochondria to Ca^2+^ activation. As with other examples, the reaction rates of OGDH and ICDH are dependent on the mitochondrial NADH/NAD ratio, because NADH is a potent inhibitor of ICDH [121]. When NADH levels are higher in the mitochondrial matrix, the enzyme ICDH is inhibited and citrate is released outside of mitochondria. The downstream enzyme OGDH is also limited, unless exogenous α-ketoglutarate is provided. Considering yet another substrate component of the TCA cycle, palmitoyl-carnitine enters the TCA cycle through acetyl-CoA, much like pyruvate, and is subsequently converted to citrate, while PDH is bypassed when using PCM as a substrate combination. Among the important take-home messages of the present analysis is the importance of recognizing the different sites of modulation by Ca^2+^ while investigating the effects of different metabolic substrates on the state 3 respiration rates characterizing the OxPhos activity. Figure 10 summarizes the hypothetical regulatory sites of Ca^2+^ ion on mitochondria bioenergetics. As described earlier and mentioned through ample evidences that Ca^2+^ accumulation occurs via MCU [103,105], DH enzymes are activated by a rising matrix [Ca^2+^] [19]. Therefore, based on our results, and as demonstrated in Figure 5, we can conclude that the regulation of mitochondrial ATP synthesis by Ca^2+^ is complex and depends upon a combination of mechanisms, including an increase in ADP and substrate supply as well as an increase in both cytosolic and mitochondrial [Ca^2+^].

### 4.5. Mechanisms That May Explain the Seemingly Paradoxical Inhibitory Effects of Higher Ca^2+^ Concentrations on Mitochondrial State 3 Respiration 

A balanced relationship between Ca^2+^ and metabolism is essential for maintaining cellular energy homeostasis. It starts with maintaining ΔΨ_m_, which is essential for balancing cellular Ca^2+^ homeostasis [122]. The alteration of ΔΨ_m_ is responsible for maintaining the fine balance between the relative rates of Ca^2+^ uptake and efflux responsible for maintaining the equilibrium between [Ca^2+^]_c_ and [Ca^2+^]_m_. This, in turn, prevents [Ca^2+^]_m_ from reaching levels that can induce an irreversible disruption of mitochondrial structure and function. As clearly seen in the present studies, the effects of Ca^2+^ on mitochondrial state 3 respiration in both heart and kidney OM are biphasic. A reduction in mitochondrial state 3 respiration in both heart and OM (Figure 5) was observed at higher [Ca^2+^]_free_. Although the stimulatory actions can be rather well explained, the underlying mechanisms for the inhibitory action on mitochondrial state 3 respiration are not well explained. Several mechanisms must be considered for explaining the observed inhibitory action (Figure 10). First, it must be considered that higher [Ca^2+^]_free_ can initiate the opening of mitochondrial permeability transition pores (mPTPs), resulting in decreased mitochondrial state 3 respiration [95]. Ca^2+^ overload can result in a reduction of the ETC activity and compromised ATP production capacity through the respiratory uncoupling and disruption of the IMM [7,123,124]. This also leads to the depolarization of ΔΨ_m_ and the inhibition of mitochondrial dehydrogenases [60,125]. Specifically, mPTP opening requires a certain threshold for matrix [Ca^2+^]_free_ concentrations >5 µM [95], and we found in the current study that the highest [Ca^2+^]_free_ in the buffer was around 1 µM, which may not be enough to attain a matrix [Ca^2+^]_free_ of >5 µM to initiate the mPTP opening. In addition, mPTP opening should significantly affect mitochondrial respiration under a leak state (i.e., states 2 and 4) [58,76,95], which was also not observed in our data. It is unlikely, therefore, that mPTP opening can explain the inhibitory effect of higher [Ca^2+^]_free_ upon mitochondrial state 3 respiration. Second, it has been proposed that Ca^2+^ and its derivatives could inhibit mitochondrial enzymes. For example, Lai et al. [125] suggested that PDH can be inhibited by a Ca^2+^ overload. Although mitochondria can strongly buffer Ca^2+^ ions that are transported from cytosol by MCU, high calcium-phosphate precipitation within the mitochondrial matrix has been implicated in reduced OxPhos activity [126,127].

### 4.6. Simple Kinetic Model Comparing the Activation and Inhibition Effects of Free Ca^2+^ on Mitochondria State 3 Respiration 

The mitochondrial state 3 respiration rates obtained in the present studies were analyzed by fitting each state 3 OCR − [Ca^2+^]_free_ curve to a kinetic model (Equation (1)) to provide a clearer representation of the effects of different [Ca^2+^]_free_ on mitochondrial state 3 respiration. As seen in Figure 9, excellent fits were obtained for most of the groups, indicating that the relationship between state 3 OCR and [Ca^2+^]_free_ can be well described by an equation that accounts for both stimulatory and inhibitory effects of [Ca^2+^]_free_ on state 3 OCR. It should be noted that only one apparent K_Ca_ value and mitochondrial matrix binding site was considered for data analysis since differentiating the activation binding constant (K_Ca_ value) from the inhibition constant did not improve the fits.

The estimated V_0_, V_max_, and apparent K_Ca_ values for heart and kidney OM mitochondria, and their corresponding confidence intervals (Figure 9), indicate that although the V_0_ and V_max_ values are much higher for the heart than they are for the kidney OM mitochondria, the binding constant K_Ca_ values are quite similar. In addition, among all of the substrate combinations used, GM was required for high levels of [Ca^2+^]_free_ to reach maximum levels of mitochondrial state 3 respiration. At these high levels of [Ca^2+^]_free_, the inhibition of state 3 respiration was already occurring for other substrate combinations. This may explain the experimental results by Panov et al. [64], who examined the effects of two different [Ca^2+^] (0 µM and 700 µM [Ca^2+^]_total_, or equivalently 0 µM and ~1 µM [Ca^2+^]_free_) in isolated heart mitochondria, but did not observe significant stimulatory effects of Ca^2+^ in the presence of the substrate combinations, AM, PM, or SUC. However, their results suggested that the state 3 OCR was stimulated by ~31–110% when using GM as a substrate combination [64]. Importantly, the present study extended the range of [Ca^2+^]_free_ used in experiments, which suggests a biphasic relationship between state 3 OCR and [Ca^2+^]_free_.

The data obtained in the present study indicate that multiple regulatory mechanisms play an important role in the inhibitory actions of Ca^2+^ (Figure 10). Specifically, the Hill coefficient for the inhibition term in Equation (1) needs to be higher than 3 to fit the experimental data, suggesting that multiple inhibitory mechanisms might be cooperative in the overall Ca^2+^ inhibition effects. It is also evident from our data that tissue-specific differences exist in mitochondrial respiration responses to Ca^2+^, and it has been found that cardiac mitochondria are more sensitive to impairment in the ETC than kidney mitochondria, as they are more sensitive to impairment of the ATP synthase and phosphate carrier [73].

## 5. Summary and Conclusions

Novel respirometry data obtained from the heart and kidney OM mitochondria of the same animal show that the heart mitochondria exhibit a > six-times higher state 3 OCR (nmol/min/mg mitochondrial protein) than that of the kidney OM mitochondria. The effects of varying [Ca^2+^] upon mitochondrial OxPhos were found to be both substrate-dependent and tissue-specific. The effects of varying [Ca^2+^] upon mitochondrial OxPhos were also found to be biphasic in both organs. The effects of varying [Ca^2+^] on mitochondrial respiration, as determined by substrate availability, were found to be different between the heart and kidney OM. When using complex-I substrate combinations, such as PM and PCM, Ca^2+^ showed more prominent activation effects in the kidney OM mitochondria compared to the heart mitochondria, as evident by percent activation of mitochondrial state 3 respiration induced by increasing [Ca^2+^]. When using SUC as a substrate in the presence of ROT, only an inhibitory effect on mitochondrial respiration was observed. The analysis of data indicates that the net effect of varying [Ca^2+^] on mitochondrial state 3 respiration is a combination of stimulatory effects on mitochondrial DH enzymes and inhibitory effects on multiple targets, including DH enzymes. As such, the net effect can be quantified by a simple empirical equation that accounts for the apparent activation and inhibition effects of Ca^2+^. Based on our results and the existing data, the fine coupling between increases in [Ca^2+^]_c_ and [Ca^2+^]_m_ is crucial to have the coordination between mitochondrial ATP production and cellular energy demands. We conclude that isolated mitochondrial studies must utilize an optimal substrate and [Ca^2+^] as defined by in vivo conditions in order to achieve physiological and optimal level of OxPhos activity and energy metabolism. These results will further our understanding of the role of mitochondrial dysfunction in the pathogenesis of diseases such as hypertension. These results are also useful for building predictive mathematical models of mitochondrial bioenergetics and regulations to study the emergent properties of cardiac and renal mitochondrial metabolic systems.

## Figures and Tables

**Table 1:** Substrate combinations that were used to measure mitochondrial respiration.

Substrate Code	Substrates *	Final Concentrations
PM	Pyruvate + Malate	5 mM + 2.5 mM
GM	Glutamate + Malate	5 mM + 2.5 mM
AM	Alpha-Ketoglutarate + Malate	5 mM + 2.5 mM
PCM	Palmitoyl-carnitine + Malate	25 µM + 2.5 mM
SUC + ROT	Succinate + Rotenone	10 mM + 0.5 µM

* The substrate stock solutions were prepared from sodium-based substrates: monosodium pyruvate, monosodium l-glutamic acid, disodium succinate, disodium malic acid, disodium alpha-ketoglutarate, and palmitoyl-l-carnitine.

**Table 2:** Conversion between total Ca^2+^ concentrations ([Ca^2+^]_total_) added to the buffer and free Ca^2+^ concentration ([Ca^2+^]_free_). Conversions were made based on Ca^2+^ chelator calculations [68]. The experimental conditions are: EGTA = 1 mM, ionic strength 0.17, pH 7.2, temperature 37 °C.

[Ca^2+^]_total_ (µM)	[Ca^2+^]_free_ (nM)
0	0
250	100
400	200
700	720
750	925
800	1230

## Data Availability

All data are presented in this manuscript.

## Abbreviation

AKGDH	Alpha-ketoglutarate dehydrogenase
AM	Alpha-ketoglutarate + malate
ANT	Adenine nucleotide translocase
BSA	Bovine serum albumin
ETC	Electron transport chain
GM	Glutamate + malate
IB	Isolation buffer
IMM	Inner mitochondrial membrane
ICDH	Isocitrate dehydrogenase
JO2	Oxygen consumption flux
MAS	Malate-Aspartate shuttle
MCU	Mitochondrial Ca^2+^ uniporter
MDH	Malate dehydrogenase
mPTP	Mitochondrial permeability transition pore
mTAL	Medullary thick ascending limbs of loop of Henle
NCE	Na^+^/Ca^2+^ Exchanger
NHE	Na^+^/H^+^ Exchanger
O2k	Oxygraph-2k
OCR	Oxygen consumption rate
OGDH	2-oxo glutarate dehydrogenase
OM	Outer medulla
OxPhos	Oxidative Phosphorylation
PCM	Palmitoyl-carnitine + malate
PIC	Inorganic phosphate carrier
PDH	Pyruvate dehydrogenase
PM	Pyruvate + malate
RB	Respiration buffer
RCR	Respiratory control ratio
ROT	Rotenone
SD	Sprague-Dawley
SUC	Succinate
TCA	Tricarboxylic acid cycle
